# An application of the edge effect in measuring accessibility to multiple food retailer types in Southwestern Ontario, Canada

**DOI:** 10.1186/1476-072X-10-34

**Published:** 2011-05-15

**Authors:** Richard C Sadler, Jason A Gilliland, Godwin Arku

**Affiliations:** 1 Department of Geography, University of Western Ontario, 1151 Richmond St, London, ON, N6A 5C2, Canada

**Keywords:** rural health, planning, food accessibility, food deserts, geographic information systems

## Abstract

**Background:**

Trends in food retailing associated with the consolidation of smaller-format retailers into fewer, larger-format supercentres have left some rural areas with fewer sources of nutritious, affordable food. Access to nutritious, affordable food is essential for good dietary habits and combating health issues such as type-2 diabetes, obesity, and cardiovascular disease. Many studies on food environments use inaccurate or incomplete methods for locating food retailers, which may be responsible for mischaracterising food deserts. This study uses databases of every residence in and every food retailer in and around Middlesex County, Ontario, Canada. Residences were geocoded to their precise address, and network analysis techniques were performed in a geographic information system (GIS) to determine distances between every residence and different types of food retailers (grocery stores, fast food, fruit and vegetable sources, grocery stores plus fruit and vegetable sources, variety stores), both when considering and neglecting facilities outside the area of study, to account for a deficiency in analysis termed the 'edge effect'.

**Results:**

Analysis of household accessibility to food outlets by neighbourhood socioeconomic distress level indicated that residents in the most distressed neighbourhoods tended to have better accessibility to all types of food retailers. In the most distressed neighbourhoods, 79 percent of residences were within walking distance of a grocery store, compared to only 10 percent in the least distressed neighbourhoods. When the edge effect was neglected, 37 percent of distance estimates proved inaccurate. Average accessibility to all food retailer types improved dramatically when food outlets adjacent to the study area were considered, thereby controlling for the edge effect.

**Conclusion:**

By neglecting to consider food retailers just outside study area boundaries, previous studies may significantly over-report the actual distance necessary to travel for food. Research on food access spanning large rural regions requires methods that accurately geocode residents and their food sources. By implementing methods akin to those in this paper, future research will be better able to identify areas with poor food accessibility. Improving identification of food desert communities is a first step in facilitating more effective deployment of food policies and programs in those communities.

## Background

The recent restructuring and consolidation of North American grocery retailers has profoundly influenced the way people access and purchase food. Many new retail developments occur in newly-developing suburbs, where land is inexpensive and transportation routes facilitate access by cargo trucks [1-3]. For rural areas, the concentration and resulting overall decline in store locations has often meant the closure of the 'hometown' grocery store [4]. The relevance of this phenomenon as a planning and public policy issue lies in its influence on population health. Since the consumption of nutritious food is an essential component to a healthy lifestyle [5], and people tend to shop near where they live [6], the spatial distribution of food retailers may influence dietary habits [7]. Various researchers have identified food deserts within the literature, a term initially coined to describe socio-economically disadvantaged areas with relatively low household incomes and poor geographical access to nutritious, affordable food sources [8].

Many studies have faults, however, including problems related to inaccurate spatial data, incomplete food retailer databases, or inappropriate spatial analysis methods. There is also a gap in the methods used to define food accessibility in rural areas. This is partly because most studies on food access consider urban areas only, and spatial accessibility in rural areas is often characterised at crude levels, like counties [4]. Other studies eschew any geographic definition of accessibility altogether [9,10]. This problem has implications for planning, since an inaccurate definition of geographic food deserts can lead to an inappropriate deployment of public policy programs intended to improve economic or behavioural food access. The purpose of this article, then, is to uncover deficiencies seen in rural studies on food environments and improve the methods used to characterise rural food accessibility. These methods will be used to provide a case study of access in Middlesex County, Ontario, Canada.

Food deserts are a well-documented but contested concept of social and physical disadvantage, with researchers both supporting [11-13] and dismissing [14-16] their existence. People with poor geographic accessibility to food manifest psychological problems (e.g. loss of dignity, forced to go against values to procure food) and create coping mechanisms for downplaying the emotional damage of dealing with inadequate access to food [11]. Even where geographic access may be adequate the prevailing cultural environment can influence the types of food and shopping that people consider acceptable [17]. Studies have indicated a disparity in food environments in less-affluent rural communities and urban neighbourhoods, where supermarkets may be scarce or stock produce of low quality [4,18]. Public health studies have indicated that well-educated, high-income populations tend to eat healthier than their less-educated, lower-income counterparts [19,20]. Healthy diets are also more expensive to attain in the absence of large-scale grocery stores [9,21]. Cross-sectional research indicates that lower obesity rates are correlated with proximity to supermarkets, while higher obesity rates are correlated with proximity to fast food and convenience stores [22,23]. The combination of these attributes translates into places where the underprivileged may be more at risk of consuming unhealthy diets. In rural areas without grocery stores, low-mobility residents may be forced to shop at variety stores, which have been shown to stock few, if any, nutritious food options [24]. For both rural and urban areas, the combined effect of poor access to food on top of individual socioeconomic disadvantage can have a deprivation amplification effect, as suggested by other researchers [25]. Therefore, it is critical that researchers use proper geospatial methods to accurately classify high deprivation areas and levels of food accessibility if policymakers and related professionals are to effectively implement programs and policies aimed at improving accessibility for the populations truly in need.

Because rural areas contain larger, less dense land masses, the GIS methods used largely in urban areas that are centred on pedestrian walkability are often inappropriate for studies in rural areas. Many rural studies have used some form of container approach, whereby the number of facilities within a polygon (postcode district, census tract, etc.) is applied generically to each polygon [26,27]. As even the smallest enumeration district in rural areas still covers a relatively large area, however, rural studies should avoid using this method to classify areas as food deserts. The use of the blanket term 'food desert' may be inappropriate, since micro-scale conditions may influence the ability of individuals to access food even where access appears to be good.

The lower population density found in rural areas means that census enumeration units, postal code areas, and network buffers for rural areas are generally larger than for urban areas. The average size of a dissemination area or DA (similar to a census block group, or CBG, in the US) in rural Middlesex County is 21 square kilometres, compared to only 0.8 square kilometres for urban DAs in the nearby city of London. Past research has used ZIP code boundaries to map socioeconomic variables and average food access, with findings suggesting the rural poor have poorer access and pay higher prices for a variety of healthy foods like fresh produce [4]. Meanwhile, other studies found little, if any, correlation between access and socioeconomic status [9]. One study found that rural supermarkets provide goods cheaper than smaller grocery and convenience stores [24]--a common result of economies of scale [28]. A study that incorporated GIS analysis by mapping socioeconomic variables and 'ground-truthed' locations of multiple food store types found that more deprived CBGs within the study area were better serviced by supermarkets, convenience stores, and discount stores [29]. These distances were contingent upon aggregation to population-weighted centroids of CBGs. This contrasts with using CBG geographic centres [15,30], which are shown to over-estimate the distance to all types of food sources when compared to population-weighted centroids [13,14,16]. This exemplifies the importance of using geographically accurate data. Yet CBGs may not accurately reflect the true accessibility for many residents within the enumeration, especially within rural areas where these span large geographic areas.

Research on food accessibility and food deserts in rural areas often considers automobile ownership as a determinant of food security, since the average distance to a grocery store is much farther and walking is not feasible [4,9]. Further work has shown that automobile ownership in general allows for a greater opportunity to shop for food as a comparison good [31]. Thus, the methods for determining food accessibility in rural areas should be framed at the scale of the automobile, since this is the only way for many rural residents to shop for food. Perversely, however, this means that low-mobility rural residents whose households do not own a car are doubly penalised, since they may be totally unable to shop at potentially distant grocery stores. The definition used to determine automobility is an important consideration. Some researchers categorise automobile ownership and access as the same variable [32], but it is important to consider that simply having access to a car may mean that a resident relies on an external source for automobility (e.g. a taxi, family member, or community member). Automobile ownership and valid driver's licences would thus be more appropriate for determining the ability of a resident to access distant grocery stores at will.

A recent, geographically proximate travel survey indicated that 90% of all trips longer than 2 kilometres are undertaken with the automobile as the means of travel [33]. In a low-density rural county, then, those with an automobile are likely to drive to access food. Some research suggests that rural residents make use of trip-chaining; for example, shopping on the commute home from work [34]. One rural study mentioned the importance of calculating accessibility from points besides the assumed home address [35], but did not posit solutions. This presumption is countered by research that shows 67 percent of shoppers started their trip from home, while 82 percent of shoppers went home after shopping [36]. Because all trips originate at some point from the home, starting from the home address is the most reasonable point of beginning for food accessibility studies, since the resident ultimately must still travel the base distance indicated in an accessibility score to reach food sources.

Methodologically, there is room for improvement in research on food accessibility in rural areas. While some urban studies have included the use of individual level data for determining accessibility [37,38], this method has not been used in rural areas. Because rural areas are sparsely populated when compared to urban centres, it is important to know where residents are living when determining food access. This study uses this method of individual accessibility to improve calculations of food access--a method which is useful for both rural and urban food accessibility research.

An additional methodological deficiency, and most central to this paper, is that studies on food accessibility in rural areas often have not considered sources outside the area of analysis, a term called the 'edge effect' [39]. In the past, this has led research to indicate food deserts at the edge of the area of analysis [29], though many of these areas may have a source of food across the border. Thus, this research needs to be studied at a geographic level of analysis that is underbounded in comparison to the corresponding food database to erase potential flaws at the edge of the study area. Recent research on this issue simulated a food environment that included outlets outside an imaginary study area. This research found inaccuracies at the edges of the study region when not accounting for external food outlets [39]. Interestingly, although more studies are making mention of potential errors caused by the edge effect, many admit fault in neglecting this concern [40,41]. This research will consider the edge effect in a real-world application to determine the effect on the accuracy of defining food access. It is hypothesised that, when not accounting for the edge effect, distances to food sources will be considerably over-reported.

While there has been some improvement in the location and characterisation of rural food stores and in the way socioeconomic distress has been examined, there is still a need to provide more rigourous methods of spatial analysis to define rural food accessibility. The issue of rural food accessibility differs from its urban equivalent, as distances to facilities are greater in rural areas and walkability frequently cannot be a consideration. Because of the dissimilar nature of rural food deserts as discussed above (i.e. the necessity of private automobiles and the greater distances travelled), and the general focus of the literature on urban areas, the methods employed in rural studies require attention.

## Methods

### Data

**Figure 1:**
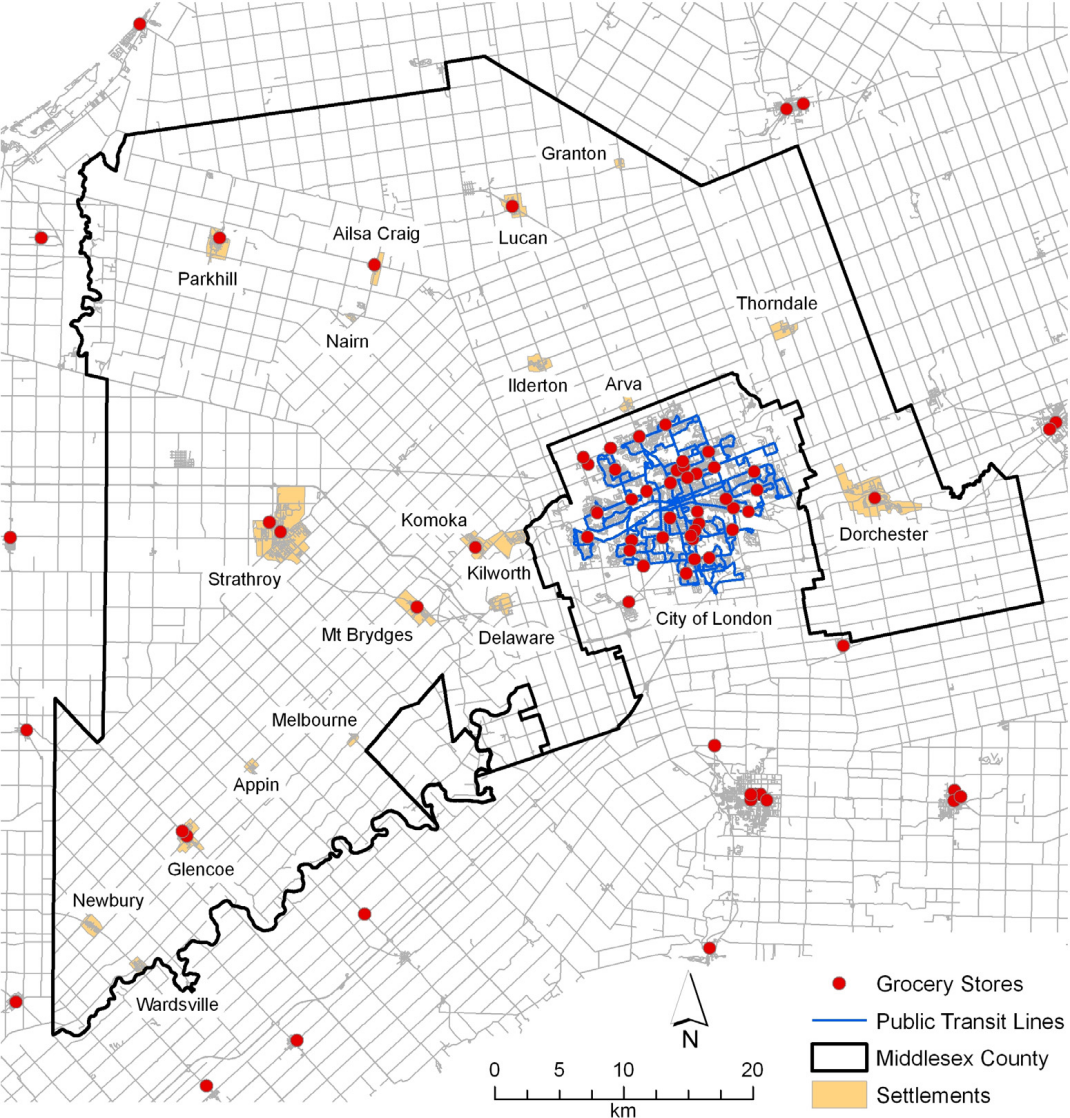
**Middlesex County, Ontario, 2010**. This map includes county boundaries, grocery stores within and outside the county, public transit lines, settlements, and roads.

This article focuses on Middlesex County (population 69,024), a rural county (as defined by the Organisation for Economic Cooperation and Development) [42], which surrounds the City of London, in Southwestern Ontario, Canada [43]. Because London is an overbounded city (and thus incorporates all of its suburbs and much agricultural land), the county has little suburban character and is largely rural. The population density is 127 people per square kilometre, a number that includes settlements with populations of 2500 or above, including Strathroy, Komoka-Kilworth, and Dorchester (Figure 1). Thus, the population density in truly rural areas is lower than this number indicates. The density of the county contrasts with the City of London (838 people per square kilometre--as calculated in a GIS). Additionally, there are no public transit services (Figure 1 shows the transit routes of the City of London--the only public transportation in the region). It is assumed that most residents must use a private automobile to access food retailers. Thus, the methods and analysis employed in this article rely on a road network when calculating distances and interpreting access thresholds--distances much larger than urban food accessibility studies discuss.

Comprehensive, up-to-date databases of retail food establishments [44] and local food producers (from the 'Get Fresh, Eat Local', or GFEL, database) [45] were geocoded to a county address file to match establishments to their exact location. All food retailers were successfully geocoded, and their locations were verified by site visits and alternative directory listings; the precision of geocoding was confirmed using 30 centimetre resolution orthophotography [46]. Despite the assurances used in this paper, previous studies have identified geocoding inaccuracies. Errors ranging from inaccurate street reference data to positional error in representation of establishments must be accounted for when relying on geocoded data [47]. Past research shows that positional errors of geocoded locations in urban locales range from 38 to 75 metres [48]. Because the scale deals with a large rural area and the average distances to food sources are high (results indicate that the average resident cannot walk to any food sources), these slight potential inaccuracies pose a considerably smaller percentage error than research conducted at a more refined, urban scale. For instance, a residence 2771 m from a variety store with a 75 m geocoding error will have a 2.7% error. By contrast, an urban residence 750 m from a variety store with a 75 m geocoding error would have a 10% error.

Given the close interchange among Middlesex County, the City of London, and surrounding areas, it was also deemed important to identify food establishments from outside the county. Addresses of food establishments from neighbouring counties were obtained using the food premises inventories provided by the health inspectors of the respective public health units for those counties [49-54], and combined with the existing food database for Middlesex-London for spatial analysis. The data in these inventories were consistent with the data for the inventory of Middlesex-London, since all inventories were created and are continually updated through legislated site visits by the respective health inspectors. In addition, local produce databases equivalent in content to the GFEL database were collected for the neighbouring counties [55-59]. Precision of all geocoding was verified against the same high-resolution orthophotography used for the initial food database [46]. Peripheral sources included all of the food sources in regions bordering Middlesex County. Analysis was run including these sources so that the lowest distance possible was returned for all residences near the boundary, a distance from the county boundary which was approximately 16 kilometres. This threshold value is not significant in itself. These sources were included to ensure that all potential facilities were considered in the analysis, thereby improving upon the methodological deficiency found in food accessibility literature that fails to account for food retailers outside the boundaries of a study area, or the edge effect. Figure 1 displays all grocery stores in and within 16 kilometres of the border of Middlesex County.

The combined food establishment database was classified into categories including 'grocery stores' (including supercentres and other grocery stores supplying a full range of fresh produce as defined by the health inspector), 'fast food' (including fast food chains and pizza take-outs), 'fruit and vegetable sources' (mostly seasonal fresh produce sellers from the GFEL database), 'grocery stores plus fruit and vegetable sources', and 'variety stores' (equivalent to convenience stores, or party stores in the US). Categories were initially defined by the Health Inspector Database, but were manually revised to better represent reality (e.g. Wal-Mart stores that do not sell groceries are not included as grocery stores). To control for potential variations in grocery store size or quality, some food basket pricing was conducted within the county. The results indicated a relative homogeneity of prices at grocery stores both within the county and when compared to the City of London. Since little variation in price existed, all grocery stores were considered in one category.

Using the categories as defined above, surfaces of accessibility for various food environments were generated. The surfaces were created by assigning distance scores to individual address points, reflecting each residence. This differs from past studies that have relied on the aggregation of data to ZIP code or county boundaries to determine food access. Areas with poor access to multiple sources of fresh produce could be considered at risk communities (given the disadvantage residents would face in procuring these goods if the establishment were to be closed). Combining areas of poor access to a source of healthy food with areas of high socioeconomic distress (to be discussed later) allowed for the mapping of communities that might be considered food deserts.

### GIS Methods

After geocoding all food establishments, analysis was conducted in the Network Analyst extension of ArcGIS 9.3 to determine individual accessibility. A current county road file [60] was used to build a geodatabase to calculate network distance. Geocoded address points for Middlesex County were obtained as the unit to which network distances would be assigned. Previous works have often relied on simplified methods for measuring access, such as the container approach [11,26,27], straight-line buffers [15,61], and network buffers [13,14,29,62]. Others have aggregated distance values to the geographical [15,16] or population-weighted centres of census enumerations [14,29,63]. Some studies employ coverage techniques of network analysis, in which the number of stores within a pre-determined distance of a residence are counted [64]. The problem with this approach is that the network considered is limited to the arbitrary distance defined by the researcher; in this instance, no reference was provided to support the thresholds created [64]. This usage of individual address points derived from land parcels to display continuous levels of access thus represents an improvement in rural food access methodology, though as discussed it has been used in the urban setting [37,38]. It is important to examine the effect of using individual address points in the rural setting, since large swaths of rural areas are unpopulated and thus irrelevant to the study of accessibility to services of any kind.

Starting from individual address points, distances were calculated to the nearest food establishments, for all food source types. The presumption that all residents shop at the nearest food retailer, however, is a flawed concept [1,65,66]. It is argued here that past research has inappropriately relied on finding the distance to only the nearest food source, and that it is imperative to consider also the second and third nearest sources. While some residents will make do with a simple provision of 'availability', research has shown that many consumers desire an alternative, or 'back-up', food retailer, whether for hedonic [65], economic [67], or cultural motives [68]. It is reasonable to suppose that the nearest food retailer may not provide the goods or customer service desired by the consumer, thus the utility of a back-up option. But even a secondary or back-up food retailer may not provide enough alternatives. Thus true 'choice' in variety of food may not occur unless the consumer has a third option. The ABCs of food access (availability, back-up, choice), then, should be considered when determining the distances to various food retailers (as opposed to simply the distance to the nearest food retailer). If the second or third nearest food retailer (back-up or choice) is too far away and the consumer refuses to shop at the nearest retailer [62], the consumer may exhibit the characteristics of living in a food desert.

### Socioeconomic Distress Index

An established strategy was employed to characterise neighbourhood-level socioeconomic 'distress' or 'deprivation'. This involved a distress index [61,69,70] modified in more recent research in the same geographical area to include key socioeconomic variables [13]. The socioeconomic distress index is an area-based measure comprised of four variables from the 2006 Canadian census: low educational attainment (proportion of adults aged 25 and over that have not graduated from high school), unemployment rate (proportion of unemployed adults who are eligible to work), lone parent families (proportion of all households with children that are headed by lone parents), and incidence of low income (proportion of households that fall below Statistics Canada's low-income cutoff for the region). These variables were chosen based on previous research, which has included identical variables for their relevance to material and social deprivation (distress), as well as issues of health and welfare [71]. Analysis was conducted at the level of dissemination area--a geographic census unit with between 400 and 700 people--since this is the smallest geographic unit for which complete Canada census data is available [43]. For each of the 134 dissemination areas (DAs) in Middlesex County, each variable was given a z-score (based on the standard deviation and un-weighted mean of the indicator). The z-scores for all four variables were summed to create a composite distress index [13,72]. Composite scores ranged from -4.44 to 8.11, with greater scores corresponding to higher levels of socioeconomic distress.

## Results

### The ABCs of Retail Food Access

**Figure 2:**
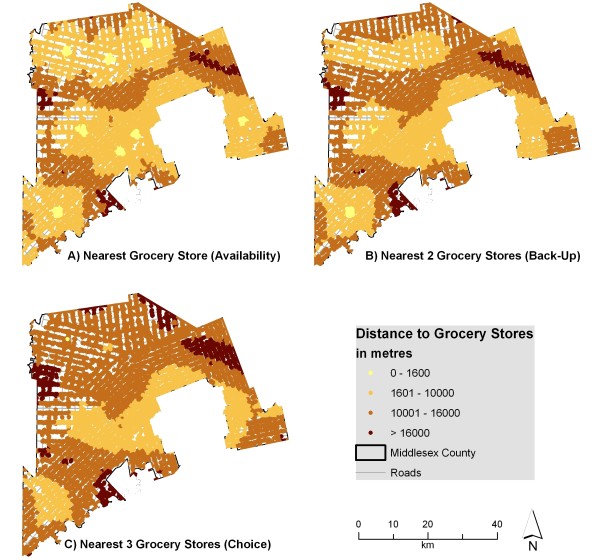
**Distance to grocery stores from individual address points when accounting for edge effect**. The network distance from individual address points to the nearest 1, 2, and 3 grocery stores was calculated and displayed using various thresholds. This indicates that for some residents, accessibility to 1 grocery store may be good, but accessibility to 2 may be problematic.

Figure 2 illustrates the distances from residences to the nearest one, two, and three grocery stores. The differences in the three maps demonstrate that the landscape of access changes dramatically for those relying on more than one grocery store; for example, areas in the north with relatively good access to one grocery store have poor access when considering two or three grocery stores. Larger settlements like Strathroy and Glencoe have two grocery stores; this is reflected in the B section of Figure 2 by the only yellow spots on the map. Yet when considering the average distance to three grocery stores, all areas in the county are over 1600 m away. Areas with a pronounced change between access to one or two grocery stores are particularly at risk for becoming geographic food deserts in the event that their local grocer closes.

**Figure 3:**
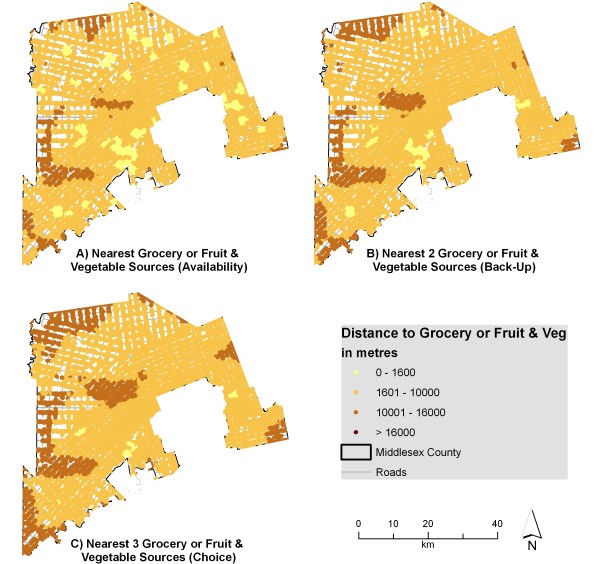
**Distance to fruit and vegetable sources from individual address points when accounting for edge effect**. The network distance from individual address points to the nearest 1, 2, and 3 fruit and vegetable sources was calculated and displayed using various thresholds. These sources include both grocery stores and seasonal produce stands classified as fruit and vegetable sources.

Figure 3 illustrates the distances to multiple sources of fruits and vegetables. Parts of the county can access three fruit and vegetable sources within 1600 m, suggesting that these residents could walk to all three. Despite this, many fruit and vegetable sources include local produce stands which may only operate seasonally or only provide a few varieties of fresh produce. The results indicate that residents tend to have better access to fruit and vegetable sources in general than to grocery stores. Recent research, however, indicated that a considerable majority of survey respondents (77 percent) visited 'major retailer stores' as their main food shopping source [73]. Research has also shown that 93 percent of US food retail store sales are made in supermarkets or grocery stores, 4 percent of sales are made at convenience (variety) stores and 3 percent of sales are made in specialty food stores [74]. These specialty food stores are equivalent to the fruit and vegetable sources presented in this research. This research demonstrates that most people will do the bulk of their shopping (both in frequency and dollars spent) at grocery stores rather than at local produce stands or variety stores.

**Figure 4:**
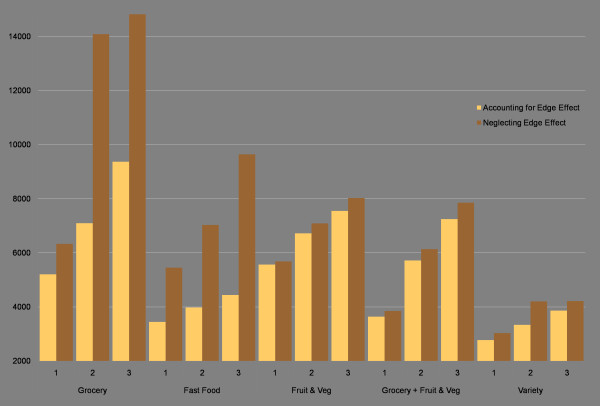
**Average distance (metres) to nearest 1, 2, and 3 food sources by type**. This figure indicates the distances to various food sources when accounting for or neglecting the edge effect. In every pair, the average distance to food sources is greater when neglecting the edge effect, since certain food sources are omitted from analysis. Paired t-tests indicate that the difference between distances when accounting for or neglecting the edge effect is significant for all food source types.

Figure 4 reports the average distance to the nearest one, two, and three food source types on the left side of each pair. There is a considerable difference in distance between the nearest one and the average distance to the nearest two or three of any type of food retailer, demonstrating that in rural areas, accessing a 'back-up' food retailer may require travelling a much farther distance.

### Accounting for the Edge Effect

Neglecting facilities outside the county boundary causes inaccuracies in the apparent distances needed to reach all food types. The values on the right of the paired graphs in Figure 4 show the distances calculated for food store types when neglecting sources outside the study area. Particularly for grocery stores and fast food, the distances are highly over-reported when neglecting external sites. For example, the actual average distance to a fast food location in Middlesex County is 3448 m. Were the edge effect not considered, the average distance would be 2001 m *farther *(the value of the error), or 5449 metres total, an error of 58%. Paired t-tests were run, and statistically significant values were found for every pair (p < 0.01). Additionally, in all cases the standard errors for each pair do not overlap.

**Figure 5:**
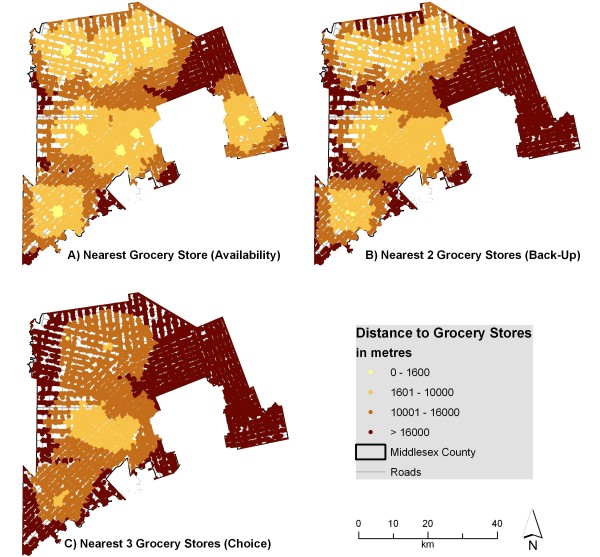
**Distance to grocery stores from individual address points when neglecting edge effect**. The network distance from individual address points to the nearest 1, 2, and 3 grocery stores was calculated and displayed using various thresholds. When compared to Figure 2, this indicates large apparent differences in accessibility when neglecting the edge effect.

**Figure 6:**
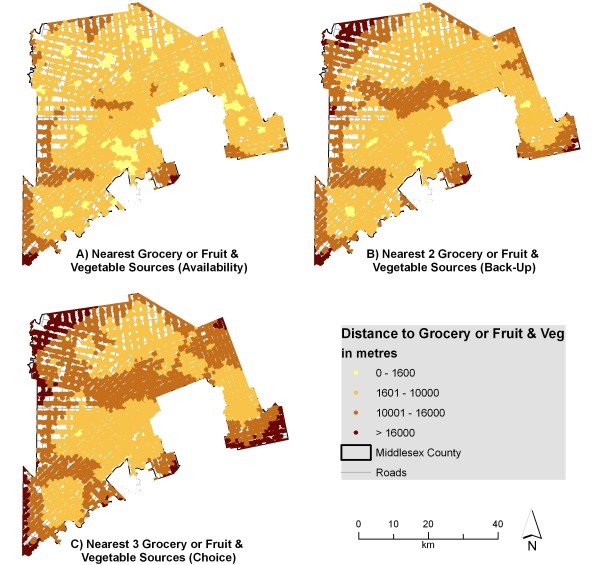
**Distance to fruit and vegetable sources from individual address points when neglecting edge effect**. The network distance from individual address points to the nearest 1, 2, and 3 fruit and vegetable sources was calculated and displayed using various thresholds. When compared to Figure 3, this indicates large apparent differences in accessibility when neglecting the edge effect.

Figure 4 indicates that errors in measurement exceed 1 kilometre for multiple categories, suggesting that areas with adequate access may be inappropriately labeled if the edge effect were not considered. Figures 2 and 3 would look quite different if the edge effect was not considered, with areas near the edge of the county exhibiting poor access (when in reality many grocery stores lie just outside the boundary in London or neighbouring counties). This difference is shown in Figures 5 and 6. For instance, accessibility appears to be worse in Figure 5 than in Figure 2, especially in the eastern part of the county. An inaccuracy such as this may lead to the inappropriate classification of this part of the county as a food desert, when an examination of Figure 2 demonstrates otherwise. This has implications for the usefulness of food accessibility studies that have not considered this noticeable source of error.

**Figure 7:**
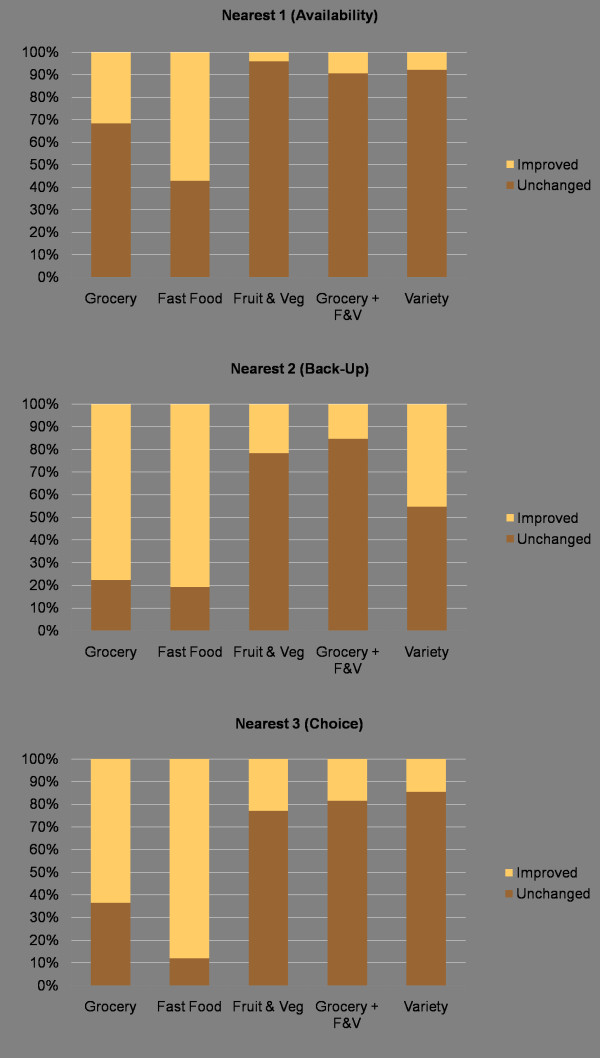
**Percent of residential addresses with improved accessibility to food when accounting for the edge effect**. When accounting for food sources outside the area of study, many address points see improved accessibility scores. The change is exemplified by the large number of addresses with improved accessibility to grocery stores and fast food, suggesting that omitting food sources will mischaracterise many neighbourhoods.

Figure 7 shows the percentage of address points whose distance values changed when the edge effect was considered. Notable is the difference when seeking the nearest 2 and 3 grocery stores, where 71 and 61 percent of address points had incorrect distance values (average errors of 5546 and 8461 metres, respectively). For calculations conducted while ignoring the edge effect, 37 percent of calculated network distances from address points increased over the original value when the edge effect was considered. The number of address points affected by neglecting external food sources is thus quite high. In addition, the average difference in distances between changed address points is high. This demonstrates that where errors occur in analysis the magnitude can be quite large.

### Socioeconomic Distress Index

The distress index scores for each census dissemination area were applied to all address points falling within the DA boundaries. The distance values to the various food sources were then cross-referenced with the individual variables in the distress index, as well as the composite distress score, and then correlation analysis (Pearson's r) was used to test for statistical associations between variables. Table 1 shows low negative correlations between distress levels and many forms of access, suggesting that there is no systematic absence of food retailers in impoverished areas. In fact, the low negative but statistically significant associations between socioeconomic distress and distance to all types of food retailers indicate that residents in the most distressed neighbourhoods travel shorter distances to reach food outlets. This finding is symptomatic of the settlement pattern of Middlesex County, from which one might infer that lower-income residents may more frequently live in larger settlements to be closer to county social services.

**Table 1:** Correlations (using Pearson's r) among distress indicators, composite distress score and accessibility to food sources

	Low Education	Lone Parenthood	Un-employment	Low Income	Composite Z-Score
Low Educational Attainment	1.00				
Lone Parenthood	**0.39	1.00			
Unemployment	**0.09	**0.15	1.00		
Low Income	**0.19	**0.27	0.00	1.00	
Distress (Composite Z-Score)	**0.67	**0.73	**0.50	**0.60	1.00
Nearest Grocery Store	**-0.16	**-0.29	**-0.22	0.02	**-0.26
Nearest 2 Grocery Stores	**-0.15	**-0.34	**-0.25	-0.06	**-0.32
Nearest 3 Grocery Stores	-0.03	**-0.24	**-0.21	-0.03	**-0.20
Nearest Fast Food	0.05	**-0.26	**-0.18	*0.10	*-0.11
Nearest 2 Fast Food	0.00	**-0.28	**-0.17	0.08	**-0.15
Nearest 3 Fast Food	-0.02	**-0.29	**-0.18	0.07	**-0.17
Nearest Fruit & Veg	0.06	**-0.17	**-0.16	-0.07	**-0.14
Nearest 2 Fruit & Veg	*0.09	-0.05	*-0.11	-0.01	-0.03
Nearest 3 Fruit & Veg	0.05	**-0.12	**-0.18	-0.04	**-0.12
Nearest Grocery or F&V	**-0.13	**-0.32	**-0.21	-0.04	**-0.28
Nearest 2 Grocery or F&V	-0.07	**-0.32	**-0.25	*-0.09	**-0.29
Nearest 3 Grocery or F&V	-0.05	**-0.28	**-0.28	-0.06	**-0.27
Nearest Variety Store	-0.05	**-0.20	**-0.12	0.00	**-0.15
Nearest 2 Variety Stores	-0.04	**-0.23	*-0.11	0.00	**-0.15
Nearest 3 Variety Stores	-0.08	**-0.24	**-0.14	-0.01	**-0.19

** - Correlation is significant at the 0.01 level (2-tailed)

* - Correlation is significant at the 0.05 level (2-tailed)

**Figure 8:**
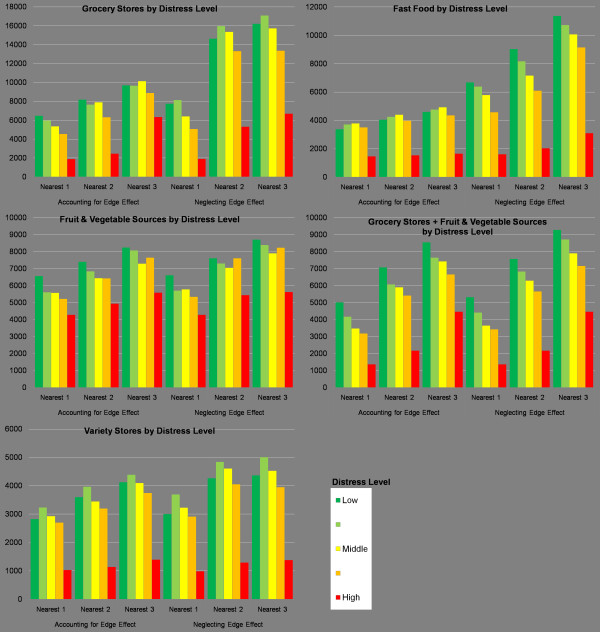
**Average distance (metres) to closest food retailers by type and distress level**. The most distressed neighbourhoods tend to have the best access to all food sources. When neglecting the edge effect, calculated distance values are always farther, which may have the effect of mischaracterising some neighbourhoods as food deserts.

**Figure 9:**
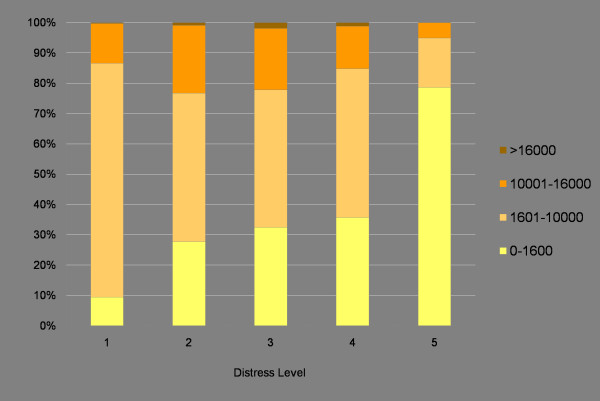
**Proportion of residences by distress level and accessibility to the nearest grocery store**. A majority of residents in the most distressed neighbourhoods are within walking distance of a grocery store. In contrast, most residents in the least distressed neighbourhoods are not within walking distance of the nearest grocery store.

Figure 8 displays the average distance travelled to food sources by residents in neighbourhoods of various socioeconomic distress levels. The distances support the assertion that neglecting the edge effect incorrectly attributes much greater distance values. They also support the finding that residents in the most distressed neighbourhoods actually travel shorter distances to all food source types. For instance, the average distance to grocery stores is less than 2 kilometres for the most distressed, while it is over 6 kilometres for the least distressed. Thus, much of the population who may have difficulty accessing a car to go shopping is actually nearer to food sources. This may, however, have adverse effects, since it also means they are much closer to variety stores (as shown at the bottom of Figure 8). In contrast to Figure 8, Figure 9 indicates the distance to the nearest grocery store by the percentage of residences within the thresholds indicated in Figure 2. In the most distressed neighbourhoods (category 1), 79 percent of residences are within walking distance of a grocery store. This contrasts sharply with the least distressed neighbourhoods, where 10 percent of residences are within walking distance of a grocery store. This indicates that not only are residents in the most distressed neighbourhoods travelling shorter distances to grocery stores, most are also within walking distance of the nearest grocery store.

## Discussion

Part of the debate over the validity of accessibility studies lies in the varying spatial methodologies employed. The increasing user-friendliness of mapping programs like ESRI's ArcGIS has allowed researchers without formal training in GIS or geography to make use of some powerful tools within the program; however, ease of use does not necessarily lead to the most geographically rigourous studies regarding the way people interact with their food environments. As discussed previously, common errors in food access studies include the use of inappropriate spatial methods (like container approaches) [15,26,27,30] and inaccurate spatial databases (such as only considering grocery stores in analysis) [29,40,41].

Based on the results presented in this paper, several issues are of concern in creating an accurate spatial representation of food access: first is the consideration of multiple, appropriately derived food source types (e.g. including sources besides grocery stores); a second concern is the consideration of food sources outside the area of interest when determining food access (the edge effect [39]); a third key concern is the methods by which distance or access to food sources is derived (including the destination that these food sources are intended to reach and the method by which this information is aggregated). This article addressed all of these methodological issues.

The incorporation of the ABC model of determining food access--and the use of multiple food types in analysis--is an important contribution to the literature. By only considering one source of food, previous research neglects the reality that many residents do not shop at the nearest food retailer [62]. This model is also important because of the potential for rural grocery stores to close due to competition from larger stores. Any closure may compel affected residents to shop at more distant grocery stores. This article has determined the differences seen when calculating the distances to the nearest two and three retailers, which in many cases is substantial.

This article improved the methods by which rural food accessibility is defined by compiling a comprehensive food database (collected from county health inspectors) that included sources outside the county boundaries. It has been shown that there is a serious inaccuracy in failing to consider external facilities when examining accessibility to food sources, since it can result in classifying areas near the boundary as having poor geographic access when they may in fact be proximate to a grocery store in the next county. This is a notable contribution, since only one article has incorporated food sources from outside a study area in a real-world study [75], and none have explicitly noted the inaccuracies by demonstrating the differences between access when both considering and neglecting outside food sources. This dearth of literature was a primary factor in developing the present paper. Finally, instead of relying on the aggregation of distance data to the nearest census division centroid, each address point in Middlesex County received its own score. If a study were conducted on individual residents, their individual accessibility could be drawn out of the GIS analysis and paired with their interview or survey responses to make direct observations between perceptions of food access and actual individual level access to food sources [76], since research suggests that accessibility is both a function of real and perceived geographic and economic access [68].

Parts of Middlesex County did have poor access to many types of food sources, though most of these areas tended to be sparsely populated. More importantly, areas of high distress tended to have better access to food sources than low distress areas. These findings suggest that many residents can access a food source by automobile within a drive of a few minutes. Given that there was no systematic absence of grocers from poorer areas, an appropriate course of action by stakeholder groups may lie in interventions beyond improving physical access.

In the communities where geographical access is potentially problematic due to high socioeconomic distress, a number of programs may help ameliorate the situation. For instance, community groups might implement shuttle programs to help those with poor access due to low mobility. One way to better understand issues related to dietary habits would be to conduct surveys or interviews with residents of these areas. If this work indicated poor dietary habits in some communities as a result of a lack of nutritious food options, smaller-scale programs like policies to encourage farm-to-school programs or farmers' markets should be considered. Farm-to-school programs have been used not only to improve community food security, but to bolster local economies and preserve farmland [77]. Farmers' markets, meanwhile, are considerably easier to implement than full-scale grocery stores, yet provide a similar effect on the price of groceries [78]. In the absence of proper funding for a full-service food retailer, these programs may serve as suitable proxies for improving the quality of life in small rural communities [63]. One small-scale campaign already underway in Middlesex County is the 'Get Fresh, Eat Local' program from which 'fruit & vegetable sources' were derived for the GIS analysis [45]. In general, programs to make healthy eating easier should be encouraged throughout the county. In Middlesex County and other areas, the geospatial methods put forth in this paper will aid in the proper identification of areas of poor accessibility to nutritious foods--areas that could be food deserts.

## Conclusions

The primary objective of this study was to improve upon the methods used to determine various forms of food access. The hypothesis was that, when not accounting for the edge effect, distances to food sources will be considerably over-reported. For all measures used (e.g. for the distance to multiple food stores, whether considering grocery stores or fast food), the results support the hypothesis. Using these methods, accessibility was combined with a socioeconomic distress index to determine whether systematic inequalities existed with respect to geographic accessibility to food sources. In this study, residences in high distress neighbourhoods had better access to all food sources, and a majority of these residences were within walking distance of the nearest grocery store.

This research has many practical implications. It not only assists current researchers by identifying the impact of common methodological pitfalls used in accessibility studies, it also has implications for policy formation. Effective evidence-based decision-making by planners and public health professionals must be based on quality evidence. This paper has presented high-quality geographic data and integrated it into several infrequently used methods to mitigate some of the errors inherent in GIS analysis for defining food access.

## Competing interests

The authors declare that they have no competing interests.

## Authors' contributions

RCS participated in study design, conducted all data analyses and drafted the manuscript. JAG funded the project, participated in study design, acquired data, assisted with the analyses, and revised the manuscript. GA was involved in revising the manuscript. All authors read and approved the final manuscript.
